# Characterizing the extrusion bioprinting process and microalgal photosynthetic efficiency in bioprinted constructs during parabolic flights

**DOI:** 10.1038/s41526-026-00669-y

**Published:** 2026-10-30

**Authors:** Johannes Windisch, Richard Frank Richter, Max von Witzleben, Finn Dani, Constantin Greil, Sarah Duin, Anja Lode, Michael Gelinsky

**Affiliations:** Centre for Translational Bone, Joint and Soft Tissue Research, Faculty of Medicine, Technische Universität Dresden, Dresden, Germany

**Keywords:** Biological techniques, Biotechnology, Engineering, Materials science

## Abstract

Extrusion-based bioprinting is a promising technology for in-space biofabrication, yet peer-reviewed process data derived in real microgravity remain rare. In the presented study, we used parabolic flights as a platform to characterize extrusion-based bioprinting of an alginate-methylcellulose bioink in short-term microgravity. Layer-by-layer printing was performed successfully at different layer distances (260–610 µm), and unsupported filament bridges of up to 13 cm were successfully printed under microgravity. We performed what is, to our knowledge, the first “green” bioprinting of microalgae-laden hydrogels under microgravity, with the cell viability of the thereby created samples exceeding 99% for *Scenedesmus* sp. and reaching 97% for *Chlorella vulgaris* on day 7 post-printing. Real-time pulse-amplitude modulated (PAM) fluorometry showed that the effective quantum yield of photosystem II remained stable across all gravitational phases in both liquid cultures and bioprinted samples while maintaining species-specific differences in non-photochemical quenching between the two species. The results of the study indicate that extrusion-based green bioprinting is functionally achievable under short-term microgravity conditions and that cultivation of the thereby created photosynthetically active structures is possible without loss of photosynthetic performance.

## Introduction

Crewed space missions are covering increasingly greater distances and longer periods of time, meaning that the crew can no longer rely on rapid general and, in particular, medical supplies being delivered from Earth^1,2^. Pharmaceutical and sterile disposable products, as well as biomedical aids compete for both mass and volume in the rocket’s storage space with other spare parts, essential items, and potential experiments. Many medical devices also have expiration dates that are relevant to the time scales of exploration missions^1,3,4^. These facts shift the ability to provide one’s own medical care, and therefore the possibility of biofabrication for medical care, from a desirable option to a potentially operationally necessary capability^5^. Among the technologies under consideration for this purpose, 3D bioprinting is proving particularly promising, as it combines digital, patient-specific image processing with cell-compatible manufacturing processes^5–7^. On Earth, this is already well established through extrusion, inkjet, and light-based approaches^6,8^.

For use in space, extrusion bioprinting is particularly feasible: the hardware is compact and robust, the control system can also be used by potential astronautical non-specialists, and, due to the absence of low-viscosity resins or liquids in the printing process, it can be operated in microgravity as an open system, both in automated and manual mode^5,9,10^. The process not only tolerates a wide range of viscoelastic bioinks, some of which are highly viscous, but also enables the production of a wide variety of different tissues or tissue models through the integration of different cell types^11–13^. The equipment can also be used as a conventional 3D printer (e.g. for thermoplastic polymers) at any time, for example for manufacturing tools with specific geometries.

However, the absence of gravity changes the balance of forces, which has a significant influence on the printing quality in (bio)printing^5,9^. Under terrestrial conditions, layers are deposited on top of each other with the support of Earth’s gravity and then held in place. In microgravity, the surface adhesion and hydrophilicity of the printing material have a much greater influence on the filament and thus also on the final shape of the manufactured constructs^14^. At the same time, microgravity provides potentially greater geometric freedom, as strands printed as overhangs or spanning cavities are not automatically brought down by gravity^7,14^. In order to establish bioprinting as a reliable capability for crewed space missions, these opportunities and limitations must be systematically investigated^5^.

Several space agencies have already announced or even flown biofabrication payloads, ranging from acoustic or magnetic field-based assembly to extrusion-bioprinting systems^5,7,9,15^. However, partly due to commercial restrictions, the published literature currently includes few freely accessible, peer-reviewed publications on extrusion bioprinting under real microgravity conditions^5,15^. Reports are often proprietary or only qualitative descriptions, making it difficult to establish general process parameters, fail-safe experimental protocols, or quality criteria for space-compatible extrusion biofabrication^7^.

For this reason, it is essential to validate the basic functionality of extrusion 3D bioprinting in microgravity, preferably before expensive space flights are carried out^16,17^. Parabolic flight campaigns are particularly suitable for this purpose^18^. In contrast to clinostats or random positioning machines, which only reorient the sample continuously and therefore do not actually reduce the acting gravity, parabolic flights generate real microgravity for short periods, in which the gravitational acceleration on the printing material is reduced in the same way as in orbit. This is important for a physical process such as extrusion bioprinting, as the behavior of the deposited strand depends directly on the actual gravitational force and not on a continuous reorientation of the sample. In these campaigns, scientists and their experiments can experience up to 30 periods of microgravity per flight, each of them lasting approximately 22 seconds and being embedded in between two hypergravity phases. This is achieved by a special flight maneuver that the pilots perform with the aircraft while the experiments are being carried out^17,19^.

In this study, we used parabolic flights as a controlled microgravity environment to investigate extrusion-based 3D bioprinting across different gravitational phases, pursuing three different aims. First, we validated the basic printing performance by testing layer-by-layer construct fabrication as well as unsupported filament bridging under microgravity. Secondly, we performed what is, to our knowledge, the first bioprinting of microalgae-laden hydrogels under microgravity conditions, followed by ground-based cultivation and viability assessment. And finally, by using pulse-amplitude modulated (PAM) fluorometry, we monitored the photosynthetic efficiency of microalgae in real time during the complete flight, investigating cell behavior both in liquid cultures and in bioprinted constructs across all gravitational transitions. Such constructs are especially promising, since photosynthetically active microalgae are of particular interest for space biofabrication, as they can address several open questions in the life support system of space platforms, since they can be simultaneously used for oxygen production, carbon dioxide fixation, waste water treatment as well as food supply and the synthesis of pharmaceutically relevant agents^20^.

## Methods

### Biomaterial ink production for microgravity printing tests

The Plasma-Alg-MC ink was prepared as described previously^21^. In brief, alginate and methylcellulose powders were sterilized separately by autoclaving at 121 °C for 20 min. Alginate powder (3 wt%, M/G ratio 1:2, Sigma-Aldrich) was freshly dissolved in fresh-frozen human blood plasma obtained from the German Red Cross (BSD Ost, Dresden, Germany). The plasma was used as fresh frozen plasma (frozen-thawed once prior to ink preparation) and was neither heat-inactivated nor fibrinogen-depleted, since fibrinogen contributes both to cell attachment and to the rheological behavior of the ink. To minimize donor-to-donor variation, plasma from five different donors was pooled for all experiments presented in this study. After dissolution, tranexamic acid (Amchafibrin, Rottapharm, Barcelona, Spain) was added at 0.7 mg per mg fibrinogen (2.26 mg per mL plasma) to prevent fibrinolysis. Subsequently, methylcellulose powder (9 wt%, M0512, molecular weight ≈ 88 kDa, 4.000 cPs, Sigma-Aldrich) was added and manually mixed into the plasma-alginate solution with a spatula until a homogeneous paste was obtained. The mixture was then stored at room temperature for 2 h to allow the methylcellulose to swell.

### Microalgae cultivation

*Chlorella vulgaris* (CCALA 269) and *Scenedesmus* sp. (CCALA 1074) were obtained from the Culture Collection of Autotrophic Organisms (CCALA, Třeboň, Czech Republic). Cells were grown in Tris-Phosphate (TP) medium in which ammonium chloride as the nitrogen source was replaced by sodium nitrate (NaNO_3_, 0.75 g L^−1^)^22^. For pre-cultivation, a starter culture of each species was inoculated separately in 50 mL of TP medium in a 250 mL Erlenmeyer flask and maintained at 24 °C under continuous light from warm-white LED lamps (180 µmol m^−2^ s^−1^). After seven days of growth, 5 mL of culture were transferred into 45 mL of fresh TP medium for sub-cultivation. The microalgae were harvested by centrifugation at 3000 rpm and used for both bioink preparation and for PAM experiments.

### Bioink preparation for green bioprinting

For the bioprinting of *Chlorella vulgaris* and *Scenedesmus* sp., an alginate-methylcellulose bioink (Alg-MC) was prepared as described previously^23,24^. Briefly, 3 wt% alginate (Sigma-Aldrich, Germany) was dissolved in double-distilled water (ddH_2_O) and autoclaved at 121 °C for 20 min. Methylcellulose (Sigma-Aldrich, Germany) was autoclaved as a dry powder at 121 °C for 20 min. Afterwards 9 wt% MC was manually mixed into the alginate solution with a sterile spatula until a homogeneous paste was obtained and kept at room temperature for 2 h to allow the MC to swell. Finally, 100 µL of algae suspension in TP medium per gram of bioink were added and mixed thoroughly using a bioink mixing tool^25^, resulting in a final microalgae cell concentration of 5 × 10^6^ cells per gram of bioink.

### (Bio)printing

Scaffolds were fabricated using a pneumatic-driven multichannel extrusion 3D printer (BioScaffolder 3.1, GeSiM, Radeberg, Germany). The bioinks were extruded through gamma-sterilized dosing needles (d = 410 µm; Globaco, Rödermark, Germany) connected to the cartridges. Scaffolds for green bioprinting experiments were printed with three layers, a 90° shift in layer-to-layer orientation, and a strand distance of 3 mm. Printing tests were carried out according to the respective print bed designs. Printing was carried out at the ambient cabin temperature of ~20 °C, with an extrusion pressure between 80 and 120 kPa (80 kPa for layer stacking, 120 kPa for filament collapse test) and a print speed that was kept constant within each individual run. The printing speed of 18 mm/s was defined by the time of microgravity available and the strand lengths of the printing tests and was established and used for both testing and subsequent green bioprinting experiments, with just minor adjustments within ±1 mm/s. The pressure values used correspond to the parameters routinely used for the same bioink under terrestrial conditions.

Stabilization of the microalgae-laden bioprinted constructs was achieved by ionic crosslinking through a CaCl_2_-based crosslinker ink (100 mM CaCl_2_ thickened with 9 wt% methylcellulose), which was applied on top of the constructs in the following 1 g phase. The crosslinker ink was then left on the constructs until sample retrieval in the laboratory, once the plane landed (~1–2 h).

### PAM measurements

The photosynthetic efficiency of microalgae (both suspended and bioprinted) was determined using pulse-amplitude modulated (PAM) fluorometry with the stationary device MINI-PAM blue (Heinz Walz, Effeltrich, Germany, equipped with a blue LED) and the control software ImagingWinGigE (Heinz Walz). The measurement principle was described in detail by Dani and Windisch et al.^22^. Briefly, microalgae-laden scaffolds were first incubated in the dark for 5 min, followed by repeated application of saturation light pulses for 300 s in 20 s intervals and measurement of the resulting fluorescence emission. Based on these recordings, we calculated the effective photosystem II quantum yield Y(II), which we used here as a readout for photosynthetic efficiency. Y(II) thereby quantifies the fraction of absorbed light used for photochemistry by PSII. To account for possible variations across different regions of the scaffolds or liquid cultures, five areas of interest (AOIs) were defined at fixed positions for each sample and averaged for the final value. For each condition, three independent samples were selected and measurements were carried out for each. For the in-flight measurements, the measurement interval was set to one measurement per second, enabling live monitoring of photosynthetic parameters during the transitions between g phases.

### Live/dead analysis

Cell viability of the microalgae in the bioprinted scaffolds was examined by fluorescence microscopy, following the general approach described previously^22^. In short, non-viable cells were labeled with SYTOX™ Green nucleic acid stain (Thermo Fisher Scientific, USA), whereas viable cells were detected via their chlorophyll autofluorescence. Images were acquired with a BZ-X800 fluorescence microscope (Keyence, Japan). Semi-automatic determination of the areas occupied by living and dead cells was carried out in ImageJ (Fiji, version 1.52p) to obtain quantitative viability values. Viability was defined as the ratio of the area of living cells to the area containing all cells. Three samples were imaged at three different positions each.

### Statistical analysis

Statistical analysis was performed using GraphPad Prism (version 10.0, GraphPad Software, USA). Data is presented as mean ± standard deviation (SD). All photosynthetic parameters and the regulated dissipation analysis were analyzed using two-way ANOVA, followed by Tukey’s multiple comparisons test for pairwise comparisons between groups. Significance levels are indicated as */#*p* < 0.05, **/##*p* < 0.01, ***/###*p* < 0.001, and ****/####*p* < 0.0001.

### Campaign overview and experimental timeline

**Fig. 1:**
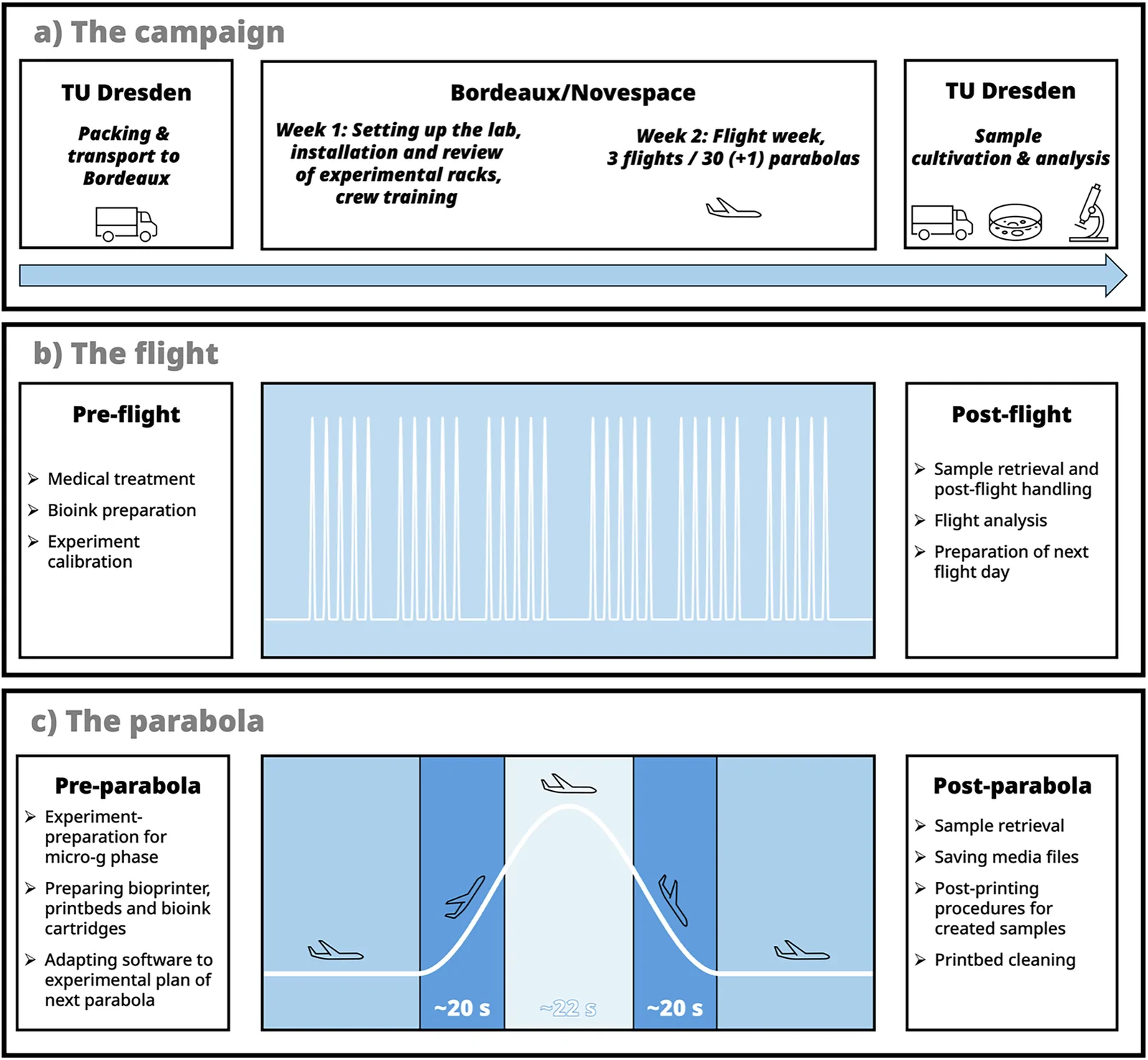
Overview of the parabolic flight campaign. **a** Transport of the experimental setup from Dresden (Germany) to Bordeaux (France) and return. **b** Flight-day timeline with pre-flight preparations, six in-flight parabola groups (five parabolas each), separated by 5–8 min breaks for experimental adjustments, followed by post-flight sample processing. **c** Altitude and g-profile of a representative parabola, comprising pull-up hypergravity (∼1.8 g, dark blue), microgravity (∼22 s, ∼10^−2^ g, light blue), and pull-out hypergravity (∼1.8 g, dark blue). Stable 1 g segments before and after the parabola (normal blue) were used for preparation and sample retrieval.

The parabolic flight campaign was conducted in Bordeaux in cooperation with the DLR Space Agency and the flight operator Novespace. As shown in Fig. 1, the campaign comprised two weeks: one week for experimental setup, safety review and installation aboard the aircraft, followed by the flight week itself. Three flights were conducted during that second week, each consisting of 31 parabolas (one acclimatization parabola followed by 30 experimental parabolas). On each flight day, samples were prepared, and experiments initialized prior to boarding. After landing, samples were retrieved, and a flight evaluation was conducted to adjust parameters for the following day.

Each parabola comprised a sequence of gravitational phases: horizontal flight at 1 g, a pull-up maneuver at ~1.8 g, a 22-second microgravity phase (µg) initiated at a climb angle of ~50°, followed by a second hypergravity phase (∼1.8 g) during the pull-out, before returning to steady-state 1 g flight.

The 30 experimental parabolas during such a flight are divided into six groups of five parabolas with 5 or 8 min-long breaks between the groups. Each parabola group was used to perform specific printing experiments with different setups, while the breaks between the groups were used to modify the experimental setup as needed.

The available microgravity time per parabola (∼22 s) and Novespace’s safety requirements constrained both the rack design and the experimental structure. Each experiment run had to be completed within a single microgravity window, and results had to be secured without manual intervention before the subsequent hypergravity phase (∼1.8 g). The rack and all experiments had to withstand the forces of all g phases and the constant transitions between them, without interfering with other experiments on board or posing a safety risk to passengers or the aircraft.

Based on these requirements, a modular experimental rack was designed and manufactured to enable safe and stable execution of three-dimensional bioprinting experiments with a common Earth-bioprinting system in microgravity.

### Experimental rack design and assembly

**Fig. 2:**
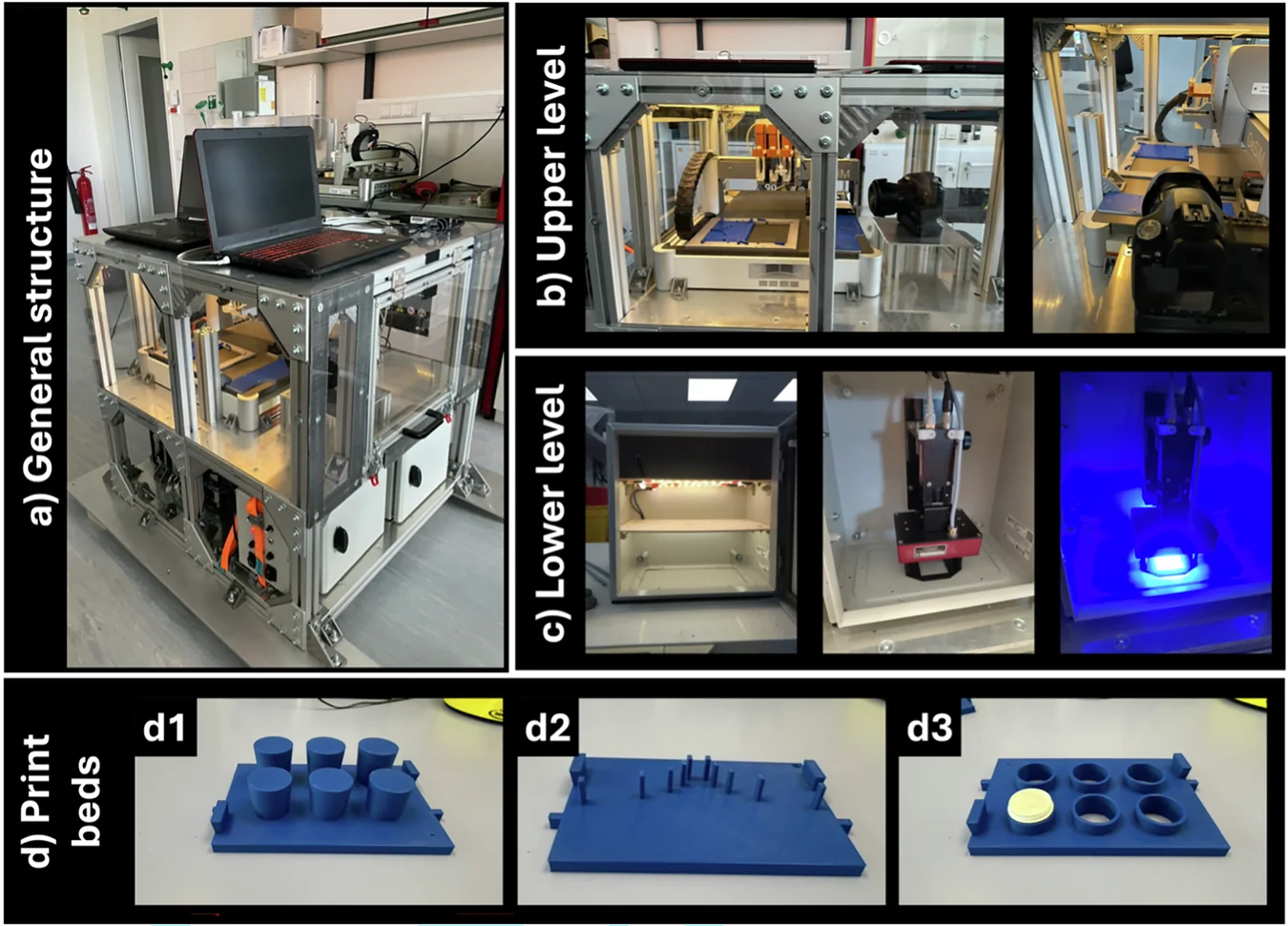
Experimental rack design and print bed configurations. **a** Fully assembled rack, later mounted to the aircraft floor. **b** Internal layout with an upper deck housing the bioprinter (GeSiM BioScaffolder 3.1) and monitoring equipment, and **c** the lower deck accommodating the pneumatic control unit (F-Box), a PAM fluorometry cabinet (lower level, middle and right), and an LED-illuminated storage cabinet to maintain algae samples between experimental runs (lower level, left side). **d** The three print bed types (printed with PLA filament) used: **d1** Platform print bed with six circular platforms for layer-by-layer construct fabrication, **d2** Bridge print bed with pillar pairs for filament collapse tests, and **d3** Jar print bed with circular openings for microalgae-laden construct fabrication for post-flight cultivation.

The experimental rack, the build-up of which is pictured in detail in Fig. 2, was designed and constructed in accordance with the minimum specifications defined by Novespace. A key requirement was structural stability sufficient to withstand the repeated transitions between gravitational phases (1 g, 1.8 g, and µg), as well as potential turbulences, hard landings, or any other loads arising from unplanned flight maneuvers including emergency situations. The main frame consisted of 45 × 45 mm Bosch aluminum profiles and was mounted to the aircraft floor via two base bars (EN-AW 6082), designed to sustain limit loads of up to 9 g. The rack measured 1168 × 1056 × 850 mm (L × W × H) with a total weight of 198 kg when fully equipped and a maximum power consumption of 7.5 A (fused at 10 A).

The internal architecture comprised three functional levels. The central main level incorporated the 3D bioprinter (GeSiM BioScaffolder 3.1; 600 × 500 × 400 mm, ~27 kg), a pneumatic extrusion-based printing system equipped with a UV pen (365 nm) for photocrosslinking, as well as all components required for successful microgravity experiments, including a digital single-lens reflex camera (Canon EOS 50D) equipped with a telephoto lens and a USB webcam (Microsoft LifeCam Studio) for real-time experiment monitoring and analysis. The printer area was thereby enclosed by a custom-built housing (845 × 845 × 500 mm) consisting of UV-protective polycarbonate panels mounted onto the primary structure. These panels blocked the UV radiation generated by the printer’s UV pen and contained any bioink within the printing compartment. This compartment was accessible through two lateral doors fitted with aluminum hinges, toggle latches, and sealed with foam rubber and silicone. Microswitches at the doors automatically cut power to the UV pen when opened.

The lower level of the setup contained a pneumatic control unit (F-Box, GeSiM; 400 × 380 × 300 mm, ∼14 kg, ∼120 W), which regulated the air pressure required for the use of the printer. Pressure was supplied by an electrically operated compressor unit (Aerotec Airliner Silent; 320 × 300 × 340 mm, ∼6 kg, ∼300 W, max. output 6–8 bar, 68 dB), fastened on an additional aluminum base plate (406 × 555 mm, total weight ∼10 kg) positioned next to the main rack. Additionally, the lower level had two IP66-rated steel cabinets (each 300 × 300 × 200 mm in size). The right cabinet served as a storage unit equipped with warm-white LED illumination for short-term maintenance of photosynthetically active microalgae samples stored away between experimental runs. The left cabinet housed the PAM fluorometer for quantifying photosynthetic efficiency of microalgae across different gravitational phases. The PAM was height-adjustable via a gear rack mechanism, with cables guided through a brushed 10” rack outlet at the cabinet top.

On the top plate of the rack two laptop computers, dedicated to bioprinter operation and photosynthetic efficiency monitoring, were mounted and held in place by Velcro fasteners.

### Experiment designs and implementation

All printing experiments described in the following sections were performed using biomaterial inks based on an alginate-methylcellulose (Alg-MC) mix (3 wt%,9 wt%), previously developed by our lab for bioprinting applications^24^. For the basic printing characterization experiments, a plasma-based Alg-MC formulation (Plasma-Alg-MC) was used, consisting of 3 wt% alginate dissolved in human blood plasma with 9 wt% methylcellulose^21^. For the green bioprinting experiments, a water-based Alg-MC bioink laden with green microalgae was used^23,24^. In addition, a crosslinker ink consisting of 100 mM CaCl_2_ thickened with 9 wt% methylcellulose was used for post-printing stabilization of the constructs.

Three different print bed types (Platforms, Bridges, Jars, Fig. 2d1–d3, accessible via repository https://doi.org/10.5281/zenodo.19367148) were designed to characterize different aspects of extrusion-based bioprinting under microgravity. A shared frame for all print beds was attached to the printer using velcro fasteners, holding the beds in defined positions. The frame provided space for two base plates serving as print bed carriers. These base plates could be changed via a puzzle-like mechanism and were locked in place during all g phases by movable switches on all sides of the frame, that could be rotated over the print beds. All print beds were designed so that the main printing area was in the focal plane of the camera mounted opposite the printer. Print beds were designed using SolidWorks (Dassault Systèmes) and fabricated from PLA by FFF 3D printing. PLA was thereby used as a structural material only. For the experiments including live cells the bioink was deposited into separate sterile, sealable jars made of polypropylene and the constructs were removed after landing for cultivation in 12-well plates, so the cells did not come into contact with the PLA support. The blue color of the material enhanced the contrast between printed (bio)ink and print bed, supporting reliable evaluation of the photographic recordings.

### Layer-by-layer construct fabrication

For general printing tests, small platforms were used (Fig. 2d1). Each print bed contained six circular platforms (diameter = 30 mm) arranged in two rows of three. To ensure good image analysis, the rear row was elevated by 5 mm compared with the front row, resulting in platform heights of 25 and 30 mm. This and the 40 mm center distance of the platforms located within the same row kept all platforms within a reasonable range for camera evaluation.

To test whether the printer could operate under µg conditions, i.e. whether a layer-by-layer deposition approach still works when gravity no longer helps to keep the upper layer on top of the previously extruded one, as well as to test whether the adhesion of the bioink together with the downward force during extrusion is sufficient to build a stable connection within the construct, four-layer constructs as 8 × 8 mm^2^ square frames (four strands per layer, see Supplementary Information SI1) were printed at layer distances of 260, 310, 410, 510, and 610 µm, manually adjusted in the G-code. Given a nozzle diameter of 410 µm, the larger layer distances (510 and 610 µm) exceeded the strand diameter, meaning that layer adhesion would have to solely rely on the stickiness of the bioink and the momentum given to the strand during extrusion, rather than the support through gravitational forces. The printing process was recorded by the camera, and the layer adhesion was evaluated qualitatively from the resulting images. A layer was considered successfully deposited when it remained attached to the underlying strand without visible detachment.

### Filament bridging in microgravity

Based on the filament collapse test firstly described by Ribeiro et al.^26^, we designed an advanced version with substantially larger gaps (8, 16, 32, 64, 128 mm) (Fig. 2d2), as we hypothesized that under microgravity much larger spans could be bridged. However, the required gap lengths would no longer fit into a reasonable camera field of view and would also bring the printer close to its technical limits for movements in x direction. Therefore, the gaps were arranged one behind the other, increasing in width towards the camera and as such forming a V-shaped arrangement of rectangular pillars on the print bed when viewed from above (CAD visualization in the Supplementary Information SI2). The rectangular pillars served as start and end points, respectively. This arrangement allowed for the analysis of each gap while continuously printing over all gaps within one parabola (∼22 s).

Filament collapse tests were initiated with constant extrusion at the smallest bridge. Once the strand adhered to the first pillar, the print head moved at a constant speed above all gap sizes while extruding the strand. To avoid strand ruptures or complications with (re-)attachment, which would have required manual intervention and prevented the completion of all bridges within a parabola, the material extrusion continued along the entire path in a zigzag, including the sections outside the defined gaps. A bridging attempt was rated successful when the strand connected both pillars across the full gap while remaining suspended, i.e. without contacting the print bed in between. Evaluation was performed qualitatively from photographic recordings, as the bridges were destroyed during the subsequent hypergravity phase (1.8 g). After each parabola the group analyzed the material behavior during the run (i.e. was too much or not enough ink deposited, did the ink stick to the needle, was the contact to the surface of the first pillar good enough?), and in the break between two parabolas the results were evaluated, the print bed was cleaned, and the printing speed and pressure were optionally adjusted for the next run.

### Microalgae-laden construct fabrication and post-flight processing

Samples intended for post-flight cultivation were printed into small white jars with sealable lids. The lid was removed before printing, and the open jar was inserted into the jar print bed, which consisted of a base plate with six holes at identical positions as the platforms. Each hole had an inner width of 25.5 mm and was therefore 0.5 mm narrower than the outer width of the jars, so the jars could be pressed into the opening and remained fixed during printing, independent of the g phase.

For 3D bioprinting into the jars under microgravity, small three-layer 6 × 6 mm^2^ grid structures in a window design (square frame with alternating one horizontal or vertical central strut each second layer, five strands per layer) were printed directly onto the bottom of the open jars. The reduced layer count compared to the basic printing tests reflects the additional inner strands and was chosen to keep the printing within the 22 s microgravity window. After the subsequent hypergravity phase, the rack door was opened and a crosslinker ink consisting of 100 mM CaCl_2_ thickened with 9% methylcellulose was applied manually via a 2 mL syringe onto each construct. Diffusion of Ca^2+^ from the crosslinker ink into the bioink led to crosslinking and stabilization of the cell-laden construct. After the addition of the crosslinker ink, the jar was closed with a screw cap, sealed with parafilm and stored in a dedicated rack. Back on ground, the jars were transported into the lab at Novespace and opened under a sterile working bench. Here, the constructs were taken out of the crosslinker ink, washed with ddH_2_O and transferred into cell culture medium, in which they were transported to the lab in Dresden, finally transferred into a 12-well plate and, after adding cultivation medium, cultivated for five additional days under warm-white LED lamps (180 µmol m^−2^ s^−1^) at 24 °C before being analyzed for cell viability as well as photosynthesis efficiency.

## Results

### 3D printing of an Alg-MC biomaterial ink in microgravity

The modular experimental setup allows the flexible testing of all print bed configurations freely within each flight. In the following chapter the results of each research question will be presented and discussed individually.

### Layer stacking at varying layer heights

**Fig. 3:**
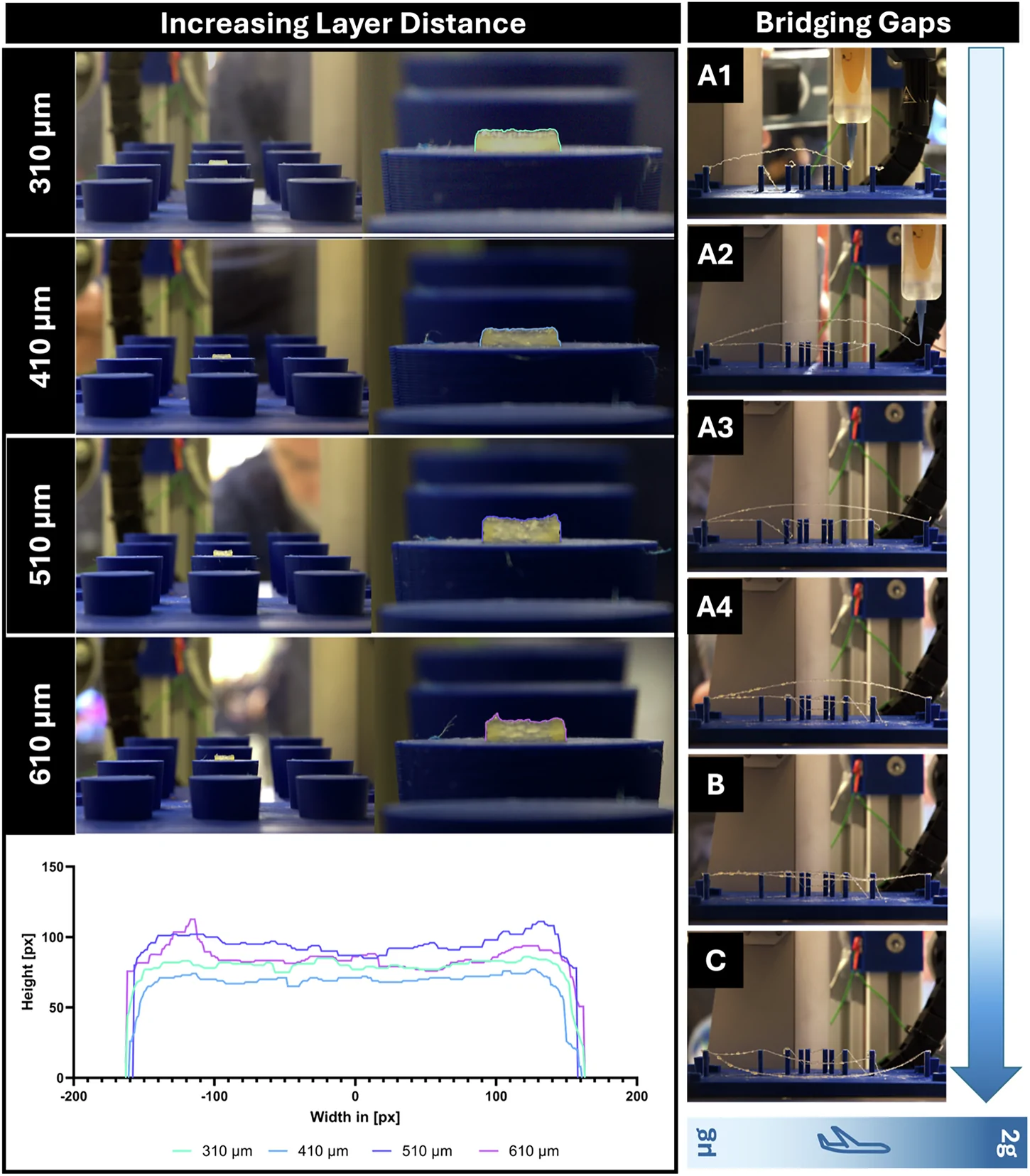
Printing performance in microgravity. Left: Layer-by-layer deposition of four-layer grid constructs printed at increasing layer spacings (310–610 µm). Each row shows the resulting construct completed within a single microgravity phase. Constructs were successfully printed at all spacings, including 510 and 610 µm, where the layer spacing substantially exceeded the nozzle diameter (410 µm). After printing, the respective height profiles of the constructs were analyzed and plotted against each other for comparison. Right: Filament bridging in microgravity with a 13 cm span between start and end pillars. **A1**–**A4** Bridge deposition from left to right, showing the pronounced upward deflection of the strand after extrusion. **B** Completed 13 cm bridge. **C** Bridge collapse during the subsequent hypergravity phase.

Four-layer grid constructs were successfully printed with layer distances ranging from 260 to 610 µm (Fig. 3, left side). Given a nozzle diameter of 410 µm, the two largest layer distances (510 and 610 µm) exceeded the theoretical strand diameter substantially. Since the strand cross section in microgravity can be assumed to remain close to circular, the strand diameter approximately corresponds to the nozzle diameter of 410 µm, so that the 610 µm layer distance amounts to roughly 1.5 times the expected strand height. This means, the contact between the strands was not initiated directly due to the path of the printhead alone (by printing one strand more or less into the previous one due to a short distance), but had to rely also on the adhesion of the non-crosslinked material itself. Nevertheless, the printing worked reliably at all tested layer distances. No layers came apart and no strands were placed in the wrong position in any of the groups and the printing kept running without interruption during the whole microgravity phase. The height profiles of the constructs were analyzed over their full width (see Fig. 3) for the groups with layer distances of 310, 410, 510 and 610 µm, respectively, which were printed with the exact same printing parameters. The profiles of the fabricated constructs with the larger layer distances (510 and 610 µm) resulted in higher scaffolds than the ones printed with the smaller distances, which was expected. At the same time, the latter groups resulted in profiles with a more even plateau, not sinking in as much in the middle as the two groups with higher layer distances.

### Filament collapse test

Five parabolas were available for filament collapse tests. After the first two parabolas, which were used to find suitable pressure and printing speed settings, in all three follow up microgravity phases, gaps of up to 128 mm in length were successfully bridged during the microgravity phases (example shown in Fig. 3, right side). During the three successfully completed bridging attempts, the printing conditions were kept constant at 18 mm/s and 120 kPa. Further optimization by reducing the printing speed would have been possible, but because the microgravity window lasted only 22 s, the test print bed would not have been fully utilized. Under conditions of constant weightlessness, such an adjustment would, of course, be possible more easily. As printing into free space differs from deposition onto a surface, the extruded material which was not pressed onto the surface and remained of course unconstrained in all three dimensions, which partly resulted in irregular strand geometries depending on external forces that under terrestrial conditions would be considered marginal. Occasional needle clogging occurred, leading to strand ruptures especially in the first two parabolas, that could be partly recovered during the print session through adhesion on the pillars. A characteristic strand behavior was observed during all bridging attempts: upon exiting the nozzle, the filament consistently deflected upward, forming a slight arc before reaching the opposite pillar (see Fig. 3, right side). Nevertheless, despite this curvature, the strands remained intact, showcasing the high flexibility of the material, and were successfully anchored to the target pillar. Since no time was available for crosslinking during the short microgravity phase, all bridged structures collapsed during the subsequent hypergravity phase (∼1.8 g).

### Green bioprinting in microgravity

**Fig. 4:**
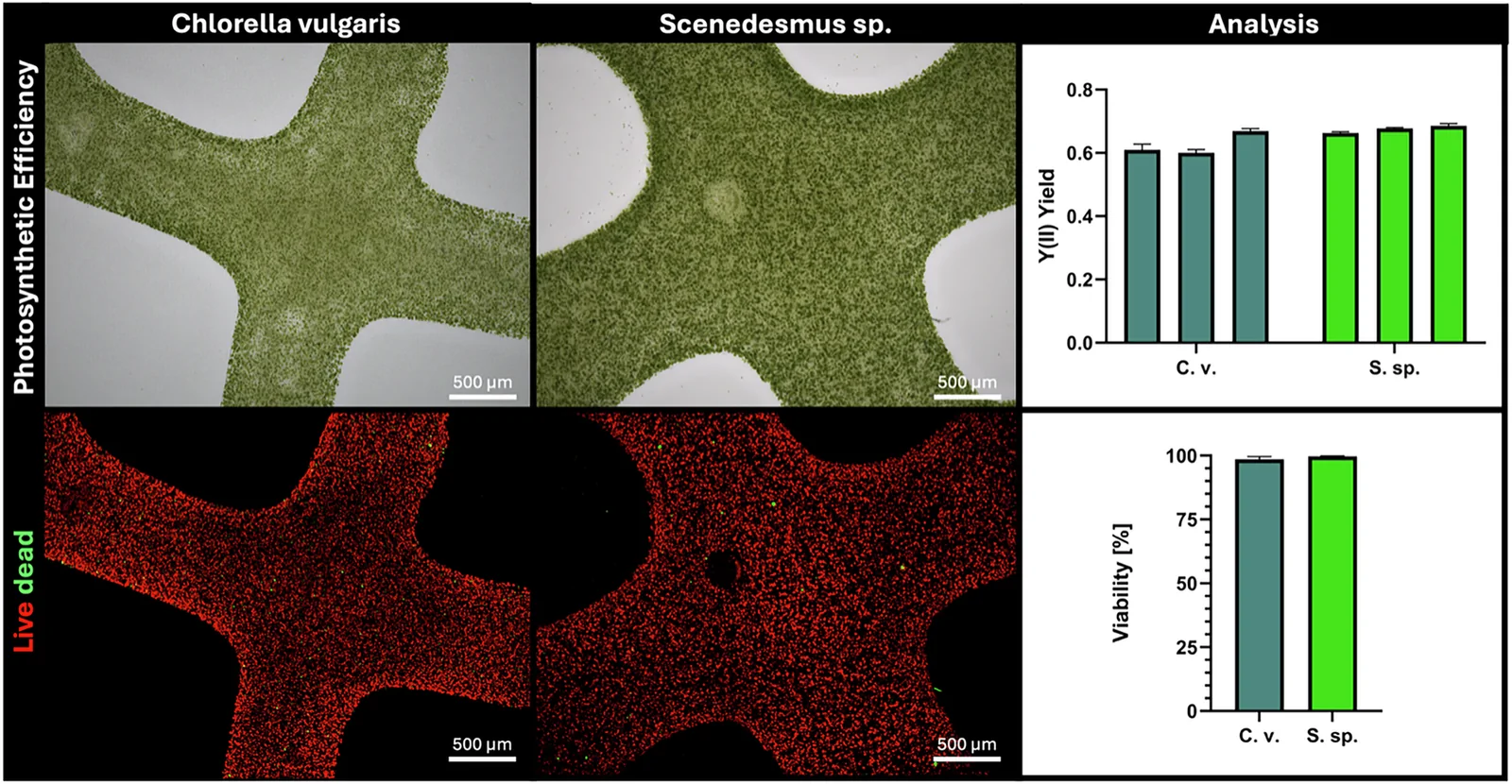
Green bioprinting in microgravity. Post-flight analysis after 7 days of cultivation. Top: Brightfield images (left, center) show dense cell populations distributed within the hydrogel strands, and photosynthetic performance assessed as effective quantum yield Y(II) by PAM fluorometry; Bottom: Live/dead fluorescence imaging of bioprinted constructs containing Chlorella vulgaris (left) and Scenedesmus sp. (center). Chlorophyll autofluorescence (red) marks viable cells, while SYTOX staining (green) indicates membrane-compromised (dead) cells; Quantitative analysis of viability (right); *n* = 3, mean ± SD; scale bars = 500 µm.

Bioinks containing either *Chlorella vulgaris* or *Scenedesmus sp*. microalgae embedded in Alg-MC blends were used to print window-shaped scaffolds in microgravity. The fabrication of the scaffolds proceeded without complications across all parabolas. The fabrication of three-dimensional multi-layer grid structures containing embedded microalgae was achieved with consistent reliability. Brightfield microscopy after 7 days of cultivation revealed dense cell populations throughout the hydrogel strands, indicating substantial proliferation (Fig. 4, upper row). It was not possible to undertake a quantitative assessment of growth rates via chlorophyll extraction due to the limited number of samples.

Live-dead imaging demonstrated high survival rates. Fluorescence images revealed a high level of red chlorophyll autofluorescence, with only a few green-stained dead cells observed across all analyzed samples. The viability of *Chlorella vulgaris* reached 97%, while that of *Scenedesmus* sp. exceeded 99% (see Fig. 4, lower row).

PAM fluorometry showed no significant differences in photosynthetic performance between species or between individual samples within each respective species. The effective quantum yield Y(II) reached high values for both species, with an average of approximately 0.60 for *Chlorella vulgaris* samples, and values slightly above 0.6 for all *Scenedesmus* sp. samples (Fig. 4, upper right).

### PAM measurements across different g-phases

As described by Kramer et al., absorbed light energy can take three competing pathways in photosynthetic organisms^27^: The photochemistry, measured as Y(II), the regulated non-photochemical quenching (Y(NPQ)), or the passive and unregulated dissipation (Y(NO)). The regulated fraction Y(NPQ) reflects active photoprotection through the xanthophyll cycle. The unregulated fraction Y(NO) includes passive thermal losses as well as fluorescence that the cell does not actively control^28^. To assess the direct effect of varying gravitational conditions on photosynthetic activity, PAM fluorometry measurements were performed continuously throughout the parabola sequences for both liquid cultures and bioprinted constructs (printed on ground 1 day prior to the flight experiment) containing either *Chlorella vulgaris* or *Scenedesmus* sp. cells.

**Fig. 5:**
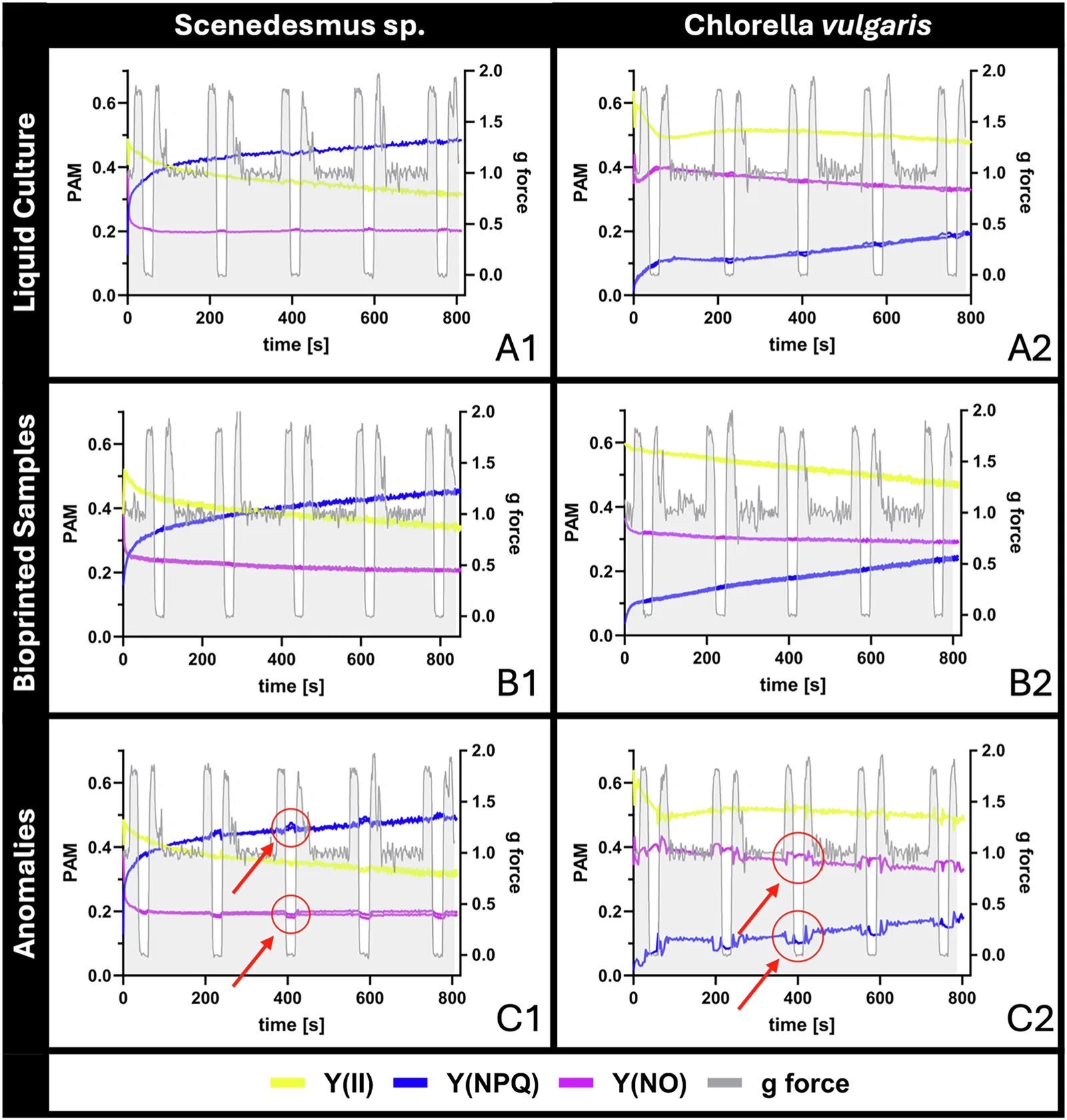
Real-time PAM fluorometry across gravitational phases during parabolic flight. Effective quantum yield Y(II) (yellow), regulated non-photochemical quenching Y(NPQ) (blue), and non-regulated energy dissipation Y(NO) (violet) are shown over time for five consecutive parabolas, with the g-profile overlaid (gray). **A1**, **A2** Liquid cultures. **B1**, **B2** Bioprinted constructs. **C1**, **C2** Representative measurements highlighting noticeable changes in Y(NPQ) and Y(NO) values for a few of the samples aligned with the according g-phase transitions, selected examples are highlighted in red.

For *Scenedesmus* sp. in liquid culture, Y(II) started at approximately 0.5 at the beginning of each parabola group and decreased linearly to approximately 0.3 over the course of five parabolas (Fig. 5A1). Y(NPQ) started at 0.3 and exceeded Y(II) after the first parabola. After that, the increase continued more slowly up to values around 0.5. Y(NO) stabilized early at 0.2 and stayed there, with only minimal fluctuations. In most samples, only minor responses to the g-phase transitions were observed.

The bioprinted *Scenedesmus* sp. samples demonstrated comparable behavior, although Y(NPQ) surpassed Y(II) only after the second parabola instead of the first (Fig. 5B1). The Y(NO) level was found to be stable at 0.2, which was consistent with the results observed in the liquid cultures. In contrast to the liquid cultures, the bioprinted samples exhibited no detectable fluctuations during the transitions to different gravitational phases.

For *Chlorella vulgaris*, the pattern differed. Y(II) was higher overall and settled around 0.5 in most samples (Fig. 5, right side). In liquid culture this plateau was reached faster. The bioprinted samples started slightly higher but declined slowly and reached 0.5 around parabola 4 (Fig. 5B2).

The partitioning of non-photochemical energy dissipation differed between species. For *Scenedesmus* sp., Y(NPQ) had been dominant while Y(NO) stayed low at 0.2. For Chlorella *vulgaris* this pattern was reversed. Y(NO) started at around 0.4 in liquid cultures and dropped only slightly, staying between 0.3 and 0.4. In the bioprinted samples it stabilized just above 0.3. Y(NPQ), in contrast, started at only 0.1 and did not exceed 0.2 by the end.

In certain samples of both species, Y(NPQ) and Y(NO) values exhibited fluctuations that coincided with the gravity-phase transitions during flight (Fig. 5, bottom row). However, the corresponding Y(II) values remained stable and were only marginally affected. The fluctuations were primarily evident in Y(NPQ) and Y(NO). For *Scenedesmus* sp., it resulted in higher Y(NPQ) values, for *Chlorella vulgaris*, fluctuations were more pronounced and appeared increased for Y(NO), with higher values recorded during both hypergravity and µg phases.

**Fig. 6:**
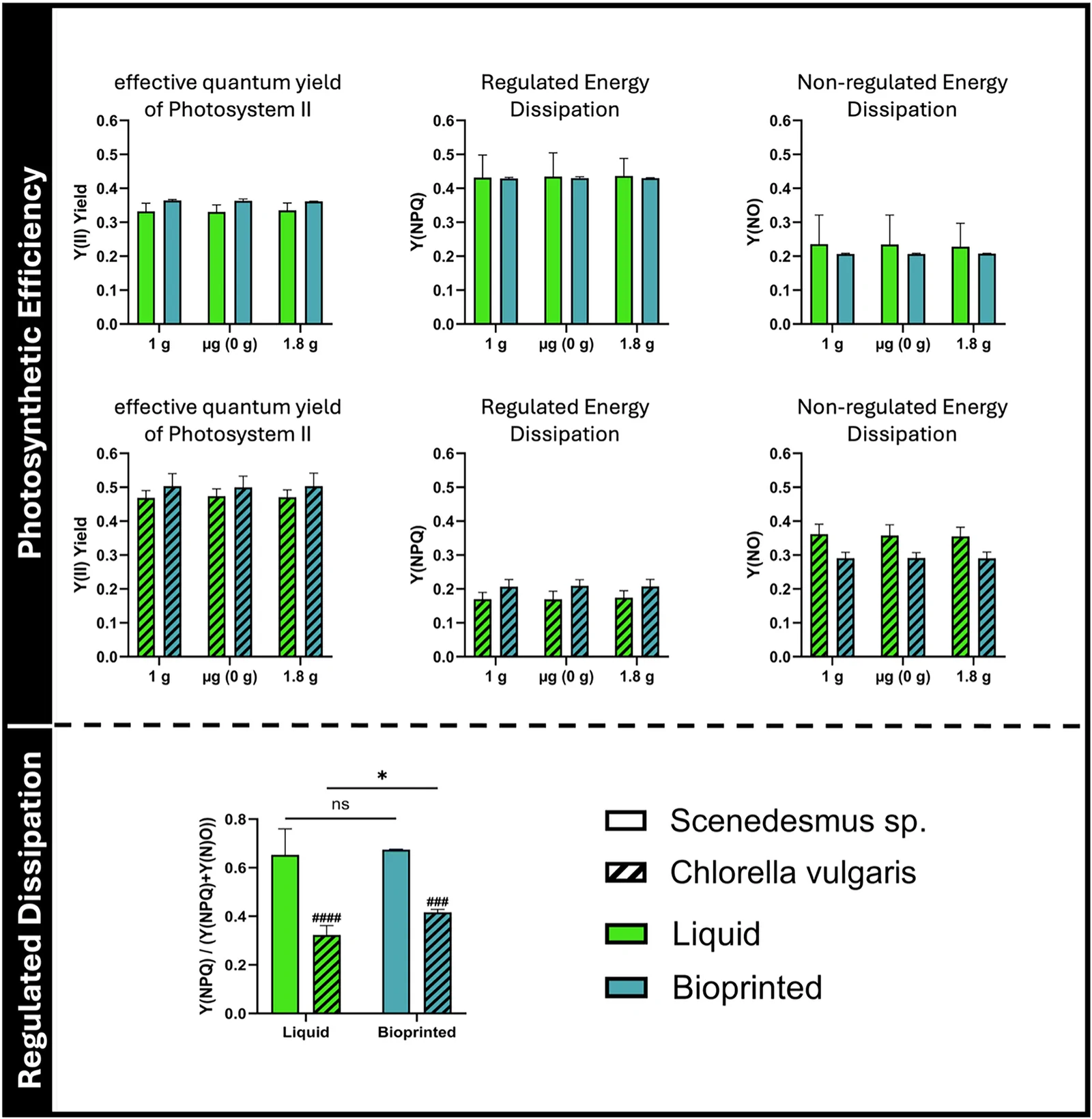
Quantitative PAM analysis across gravitational phases. Top (photosynthetic efficiency): effective quantum yield of photosystem II Y(II), regulated energy dissipation Y(NPQ) and nonregulated energy dissipation Y(NO), each averaged within the specific gravitational phases for Scenedesmus sp. (upper row, clean bars) and Chlorella vulgaris (lower row, striped bars), shown for liquid cultures (green, Scenedesmus sp. *n* = 5, Chlorella vulgaris *n* = 6) and bioprinted samples (blue, all *n* = 3). Bottom (regulated dissipation): Proportion of regulated energy dissipation Y(NPQ)/(Y(NPQ) + Y(NO)) for both species in liquid and bioprinted samples within the respective species (* equal statistics between state of samples, # equal statistics between strains; *p < 0.05, ###*p* < 0.001, ####*p* < 0.0001). all values mean ± SD.

To compare the PAM values between the g-phases, the values of each sample during parabola 4 (when the measurements reached their stable state) were averaged within every g-phase (1 g, µg, 1.8 g) and compared between said phases (Fig. 6). Y(II), Y(NPQ) and Y(NO) did not differ significantly between the three g-phases, neither for the liquid nor for the bioprinted constructs of both species, confirming that the photosynthetic parameters stayed stable across the g-transitions. To describe the species-specific distribution of the non-photochemical energy dissipation, the regulated fraction was calculated. This fraction was significantly higher in Scenedesmus sp. than in Chlorella vulgaris, both in the liquid cultures and in the bioprinted constructs. While the values thereby did not differ significantly between liquid and bioprinted samples with Scenedesmus, for Chlorella vulgaris the distribution increased in the bioprinted state.

## Discussion

During the parabolic flight campaign, layer-by-layer extrusion-based 3D bioprinting with the Plasma-Alg-MC biomaterial ink demonstrated reliable performance under microgravity conditions. The deposition of strands was found to be consistent, and the stacking of layers was successful at all layer distances that were tested, including configurations in which the layer distance exceeded the nozzle diameter of 410 µm. In the absence of gravitational force, the stability of the constructs was dependent on material cohesion, surface wetting and the downward momentum imparted to the strand by extrusion pressure. This supports the hypothesis by Van Ombergen et al. that, in space, rheological properties and print bed wettability become more significant than gravity in determining print fidelity, as gravity is effectively removed from the force balance^5^.

Prior to parabolic flight testing, inverted printing under terrestrial gravity had been proposed as a screening method for bioinks intended for use in space. Cubo-Mateo et al. introduced the approach on the assumption that a material capable of maintaining structural integrity when printed against gravity at 1 g, should perform at least equally well in microgravity^7^. Ji et al. confirmed this principle using a droplet-by-droplet deposition approach in inverted orientation^29^. The findings of this study align with this reasoning, as the Alg-MC bioink system, which had previously passed inverted printing tests on Earth, also produced stable multi-layer constructs in actual microgravity. However, it should be noted that inverted printing does not replicate all aspects of microgravity, since aspects such as altered convection or sedimentation behavior are absent or different under terrestrial conditions, regardless of the applied print orientation.

The filament collapse tests revealed additional effects specific to in-air deposition in microgravity. During the series of bridging attempts conducted, it was observed that the extruded filament consistently deflected upwards after exiting the nozzle. This was followed by the formation of a slight arc before the filament reached the opposing support pillar. Two complementary mechanisms might have contributed to this observation, with the first being that the microgravity achieved during parabolic flights was not perfectly stable. All experiments still experienced residual, fluctuating accelerations during the microgravity phase, typically in the magnitude of around 10^−2^g^30^. The magnitude of these residual forces depends on flight dynamics and weather conditions but also varies with the position of the experiment inside the aircraft. In the forward section of the aircraft, where the rack was positioned, these forces are estimated to be at the upper end of the range, according to Novespace. A hydrogel strand of low mass placed in a freely suspended state appears to be sensitive even to such small accelerations. Secondly, the phenomenon of capillary effects at the nozzle tip may have caused an accumulation of Alg-MC on the outer surface of the needle during extrusion, resulting in surface tension, which can redirect the emerging strand away from its intended trajectory. This effect occurs occasionally in terrestrial printing as well but is normally counteracted by gravity. In microgravity however, even small differences in wetting at the nozzle can become decisive for the printing accuracy^31^. Theoretically, a higher printing pressure could compensate for these problems, but in that case the volumetric flow would also increase simultaneously, complicating the parameter tuning for a successful bridging attempt drastically.

Despite this deflection, bridges of up to 128 mm were printed, a span maximum which was set by the print bed geometry, not by strand failure at longer distances. On ground, the same filament collapse test typically only includes bridges of up to 16 millimeters^26^. In our own ground-based testing with the Alg-MC system, we were not able to bridge unsupported gaps beyond 16 mm under 1 g (see also Supplementary Fig. S3), irrespective of the applied pressure and speed, while spans of up to 128 mm were achieved in microgravity. This contrast indicates that the extended bridging length is a direct consequence of the absent gravitational pull rather than of parameter optimization. In the absence of gravity, which forces a strand downwards after extrusion, the creation of overhangs and hollow structures should in theory be way more feasible, and possible even without the necessity for support baths or sacrificial materials. If successfully implemented in stable microgravity, this could enable simpler fabrication of tissue architectures that currently rely on additional utilization of sacrificial support inks, such as vascularization channels and anatomically shaped or even hollow constructs.

However, directional changes during non-contact printing and free deposition without an initial anchor point do not appear feasible. The extrusion process imparts momentum to the strand, which in microgravity caused drift unless the strand was attached to a surface or a previously deposited layer. Surface tension and the momentum given during extrusion were the dominant forces affecting the extruded bioinks when gravity was absent^5^, making a solid starting point, as well as support points every time a strand ends or changes direction, a necessity for reliable strand placement.

These results should be read against the difference between parabolic flight and orbital environments. On the International Space Station, experiments experience gravitational accelerations of potentially up to 10^−6^g, roughly four orders of magnitude lower than during parabolic flights^32^. The collapse of non-crosslinked structures during the transition to ∼1.8 g, as observed in our campaign, would not occur aboard a space station with continuous microgravity. In that sense, parabolic flights represent a worst-case testing platform. It is therefore reasonable to expect that a process succeeding under these repeated g-transitions and residual accelerations would also work under sustained microgravity, although this extrapolation still needs to be confirmed in an orbital experiment.

A direct comparison with other space bioprinting studies is limited by the scarcity of available, peer-reviewed data. Several biofabrication systems have been deployed in space, including the BioFabrication Facility operated by Redwire, the Organ.Aut device by 3D Bioprinting Solutions for magnetic levitation assembly of tissue spheroids, and autonomous Chinese bioprinting hardware^5,7,15,33^. Nonetheless, detailed process characterization data from these facilities is largely absent from the accessible literature. This is due to the fact that they either operate commercially or because the results have yet to be published^5,9^.

The only peer-reviewed study reporting extrusion-based bioprinting results from the ISS is by Klarmann et al., who used a photocrosslinkable collagen ink to print meniscus-like constructs using the BioFabrication Facility^15^. Although the fabrication of the tissue model was successful, the authors reported that its shape fidelity and mechanical stability were reduced in comparison with the ground controls. They attributed this to altered crosslinking dynamics in microgravity. In contrast, the Alg-MC scaffolds in the present study maintained their structural integrity through the microgravity and hypergravity phases without any crosslinking, relying solely on their viscoelastic properties. A direct comparison between the two studies is limited by differences in bioink composition, crosslinking strategy, and construct geometry. However, both studies confirm that extrusion-based bioprinting is technically feasible under microgravity.

The successful fabrication of microalgae-laden structures during the parabolas demonstrates the possibility of extrusion-based green bioprinting under altered gravity conditions. The printing parameters established on ground and adjusted to the framework conditions of the parabolas remained mainly the same when compared between ground and parabolic flight. It is evident that shear forces during extrusion are identical, given that they are determined by flow rate, nozzle diameter, and bioink viscosity, all of which are gravity-independent. However, a notable shift in force dynamics occurs after the strand’s ejection from the nozzle. The phenomenon of gravitational settling is not a contributing factor, and the placement of strands is instead reliant upon adhesion, wetting, and the viscoelastic recovery of the bioink^5^. Mišković et al. were able to show during their parabolic flight campaign, that stiffer viscoelastic hydrogels had a better ability to preserve their shape when enduring gravitational transitions than softer hydrogels^34^, which is consistent with our observations, that the comparatively high viscosity of the Alg-MC system can build stable constructs without gravitational support^24^.

One constraint of the parabolic flight workflow was that crosslinking could not be performed during the microgravity phase itself. Constructs were printed during the µg windows but had to pass through the subsequent hypergravity phase (∼1.8 g) in a non-crosslinked state. The needed CaCl_2_ crosslinking could only be applied after the aircraft returned to normal gravity conditions (1 g). Although the implementation of a system that would enable crosslinking during the µg phase would undoubtedly be preferable, this was not a viable option within the 22 s time window. The fact that the constructs survived the gravitational transitions in their non-crosslinked state demonstrates that the Alg-MC system provided sufficient structural integrity through its viscoelastic properties alone.

Under terrestrial conditions, microalgae are known to demonstrate a high level of tolerance to extrusion when embedded in Alg-MC bioinks. Lode et al. showed that the microalgal strain *Chlamydomonas reinhardtii* survived the printing process and proliferated well within the hydrogel, with chlorophyll content increasing more than 16 times from day 1 to day 12^23^. Additionally, Krujatz et al. confirmed growth rates of up to 70% per day at 26 °C for immobilized microalgae in Alg-MC hydrogels^35^. The viability measurements obtained here (97% for *Chlorella vulgaris* and over 99% for *Scenedesmus* sp.) correspond to or exceed the corresponding ground values. This indicates that the microgravity environment and repeated gravitational transitions did not induce additional cell damage beyond that possibly produced by the extrusion process itself. The slightly higher viability of *Scenedesmus* sp. compared to *Chlorella vulgaris* is consistent with previous observations by Dani and Windisch et al.^22^ and with reports by Moreno-Garrido, who described *Scenedesmus* sp. as particularly robust when immobilized^36^. It is more probable that this phenomenon is species-specific rather than being attributable to altered gravitational conditions.

As Boularaoui et al. emphasized, most cell damage in bioprinting occurs due to shear forces inside the nozzle during extrusion, and the extent of the damage depends on the flow rate, nozzle diameter, and bioink viscosity^37^. None of these parameters change with different gravity conditions as already described above. The shear-thinning behavior of the Alg-MC system reduces viscosity during extrusion and thereby exposes embedded cells to a lower shear stress than a Newtonian fluid of equivalent resting viscosity^24,38^. In addition, green microalgae possess rigid cell walls that provide mechanical protection^39,40^, unlike mammalian cells.

It is important to note that, in addition to the extrusion process itself, parabolic flight introduces stressors that are not present under terrestrial conditions. Such stressors include repeated gravitational transitions over a three-hour flight and aircraft-related vibrations. However, given the short duration of each µg phase (22 s), the cells were afforded sufficient recovery-time during the intervening 1 g phases and additionally during the subsequent multi-day cultivation period ahead of the analysis.

Those assumptions are supported by the real-time PAM data from the flights. Y(II) stayed stable for both species across all parabola sequences and conditions, meaning all photosynthetic processes kept running at a comparable efficiency regardless of the gravitational level. This finding is consistent with the microgravity observations reported by Balan and Stüeken, who noted that the photosynthetic efficiency yield Y(II) of *Nannochloropsis salina* and *Spirulina platensis* remained relatively stable on average during parabolic flights, despite significant fluctuations during the periods of gravity transition^41^. Whilst gravitational changes seem to impact regulatory photosynthetic processes, the photochemistry itself appears to be unaffected.

*Scenedesmus* sp. and *Chlorella vulgaris* differed in how they partitioned non-photochemical energy dissipation between the regulated and unregulated fractions. *Scenedesmus* sp. showed the expected increase in Y(NPQ) (0.3–0.5) while Y(NO) stayed low (at 0.2), indicating active routing of excess energy into the regulated quenching pathway. This is consistent with the work of Masojídek et al., who found that *Scenedesmus* possesses a larger xanthophyll-cycle pigment pool and higher quenching capacity than Chlorella^42^.

For *Chlorella vulgaris*, this pattern was reversed: Y(NO) remained stable (0.3–0.4) while Y(NPQ) stayed below 0.2. A high Y(NO) is typically interpreted as a sign that regulated quenching is not operating effectively^43^. However, Y(II) was comparable for both species, meaning that the same fraction of light was used for photochemistry. The discrepancy between the two species can be attributed to the manner in which the remaining energy was dissipated, rather than to any variation in photosynthetic efficiency per se. Quaas et al. showed that the structural basis of non-photochemical quenching is heterogeneous even among green microalgae^44^, and the lower Y(NPQ) in *Chlorella* in relation to *Scenedesmus* therefore seems to reflect a species-specific quenching strategy rather than a failure of photoprotective processes.

Both species showed fewer fluctuations during gravity transitions in their bioprinted samples compared to liquid cultures. In the *Scenedesmus*-laden constructs, Y(NPQ) overtook Y(II) only after the second parabola of the group rather than after the first, as in the liquid cultures. The *Chlorella vulgaris* bioprinted samples showed more stable Y(NO) values than *Scenedesmus* sp. samples. This could be attributed to the hydrogel matrix, which physically buffers the cells against the mechanical effects of changing gravity, whereas in liquid cultures, sedimentation and convection patterns shift with each gravitational transition. As Krujatz et al. previously demonstrated, the immobilization of microalgae using alginate protects against environmental fluctuations, which may also include temperature or osmotic shifts^35^. Our data extend this protective effect to gravitational transitions.

In samples showing more pronounced changes (Fig. 5C1, C2), those were limited mainly to Y(NPQ) and Y(NO), while Y(II) seemed unaffected. This indicates that the observed shifts were more the result of a redistribution of energy dissipation between quenching pathways, rather than a change in the overall photosynthetic capacity of the embedded cells. Non-photochemical quenching can respond to environmental changes within seconds, and it is plausible that changes in cellular physiology during gravity transitions can shift the balance of energy partitioning within the cell^45^.

This is supported by the high post-flight values for Y(II) and viability of both species indicating healthy and metabolically active microalgae (Fig. 4)^22,46^. This confirmed that the embedded cells were not lastingly impaired by the repeated gravitational changes during flight. The 1 g behavior of the system is well characterized under normal gravity conditions^22,23,38,47^, and the stable 1 g segments of each sample before and after each parabola showed no shift relative to the known ground values. Also the bioprinted samples showed no drastic changes over the course of the parabola flights, arguing against a cumulative effect of the repeated gravitational transitions. However, PAM fluorometry only provides information about the photosynthetic apparatus^48^. Normal Y(II) values after a five-day recovery period do not exclude sublethal effects on other cellular processes such as cell division, growth rate or membrane composition. Fais et al. recently demonstrated that *Chlorella vulgaris* altered its growth behavior and lipid composition under clinorotation while maintaining photosynthetic capacity^49^. The PAM data therefore confirm that the photosynthetic machinery was intact but cannot rule out other gravity-induced effects.

The overall sample number in this study was limited by the structure of the parabolic flight campaign itself: the number of parabolas was quite small, whereas the number of questions that needed to be addressed was much larger, resulting in the careful selection of only a few analyses that could be performed. Within these constraints, we focused on the readouts that give the most direct information on cell health from a small number of samples, namely cell viability, photosynthetic efficiency measured by PAM fluorometry, and a qualitative assessment of cell-population development. As a result, cell growth analysis could only be conducted qualitatively, as no samples remained for chlorophyll extraction. Also, it cannot be ruled out that the transport of the printed samples from Bordeaux back to the lab in Dresden by car may have affected the samples (~48 h). Five days of standardized cultivation back in the lab should provide a sufficient recovery window for the cells, but nevertheless this should be considered when interpreting the results. Additional experiments as part of the space-capable validation are needed and planned as part of an upcoming clinorotation study addressing these questions with larger sample numbers and controlled cultivation conditions.

Taken together, the data from this campaign show that extrusion-based green bioprinting, established originally under terrestrial conditions, also works under altered-gravity conditions without significant loss of photosynthetic activity as the most essential cell function. However, these findings are based on short-term, repeated altered-gravity conditions and their transfer to a sustained orbital environment will need to be validated in future flight experiments. This is nevertheless relevant, since microalgae are increasingly considered for use in bioregenerative life support systems on space platforms, where they provide oxygen and remove carbon dioxide^50,51^, as well as in regenerative medicine as oxygen supplier^22,52^ or as part of living building materials^53^. The presented data, together with an earlier study showing that Alg-MC based bioinks can be stored for weeks without losing printability or cell function^38^, support the feasibility of in-space biofabrication with photosynthetically active cell-material combinations.

## Supplementary information

Supplementary information

## Data Availability

The CAD models of the custom print beds used in this study are available via Zenodo at https://doi.org/10.5281/zenodo.19367148. All other data supporting the findings of this study are available from the corresponding author upon reasonable request.
