# Supplementary material for: Characterizing the extrusion bioprinting process and microalgal photosynthetic efficiency in bioprinted constructs during parabolic flights
Source: NPJ Microgravity. 2026 Oct 30;12:90. doi: 10.1038/s41526-026-00669-y (PMC13722694; doi:10.1038/s41526-026-00669-y)
Supplement: Supplementary file 1 [file 41526_2026_669_MOESM1_ESM.pdf]

# Supplementary Information

**Characterizing the extrusion bioprinting process and microalgal photosynthetic efficiency in bioprinted constructs during parabolic flights**

*Johannes Windisch, Richard Frank Richter, Max von Witzleben, Finn Dani, Constantin Greil, Sarah Duin, Anja Lode, Michael Gelinsky*

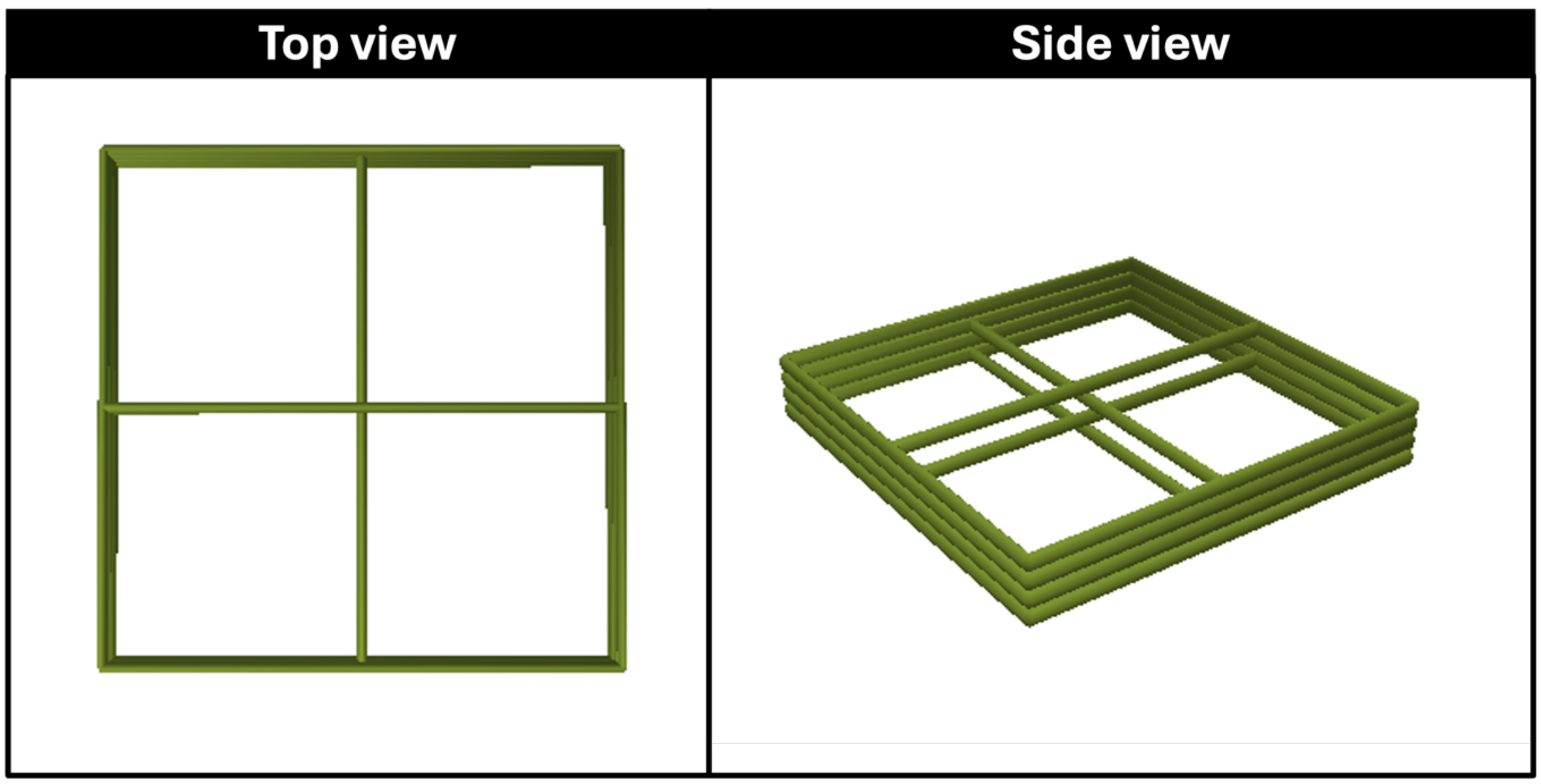


S1: Top and side view of the printed microalgae-laden constructs.

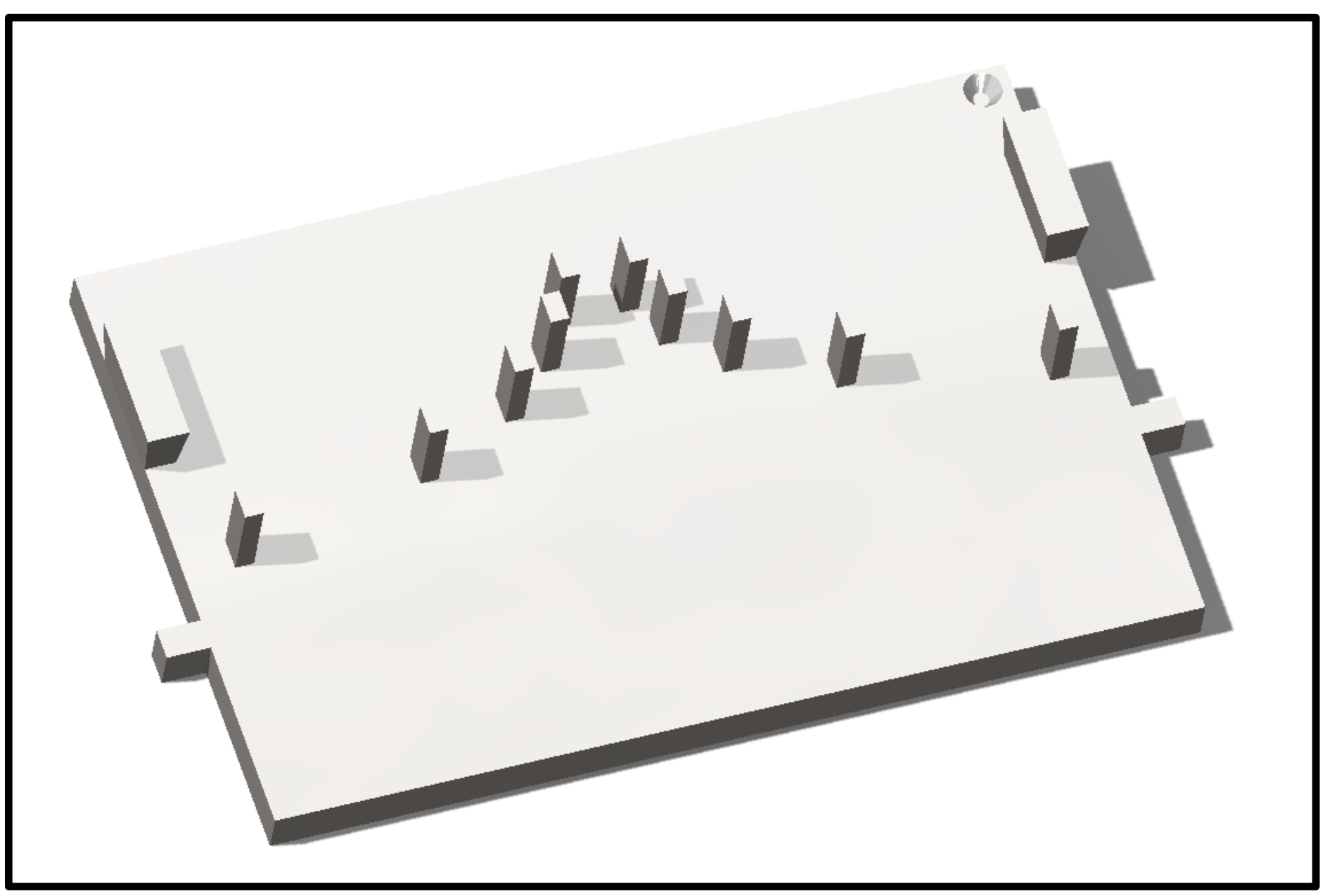

S2: Top view of the filament collapse print bed.

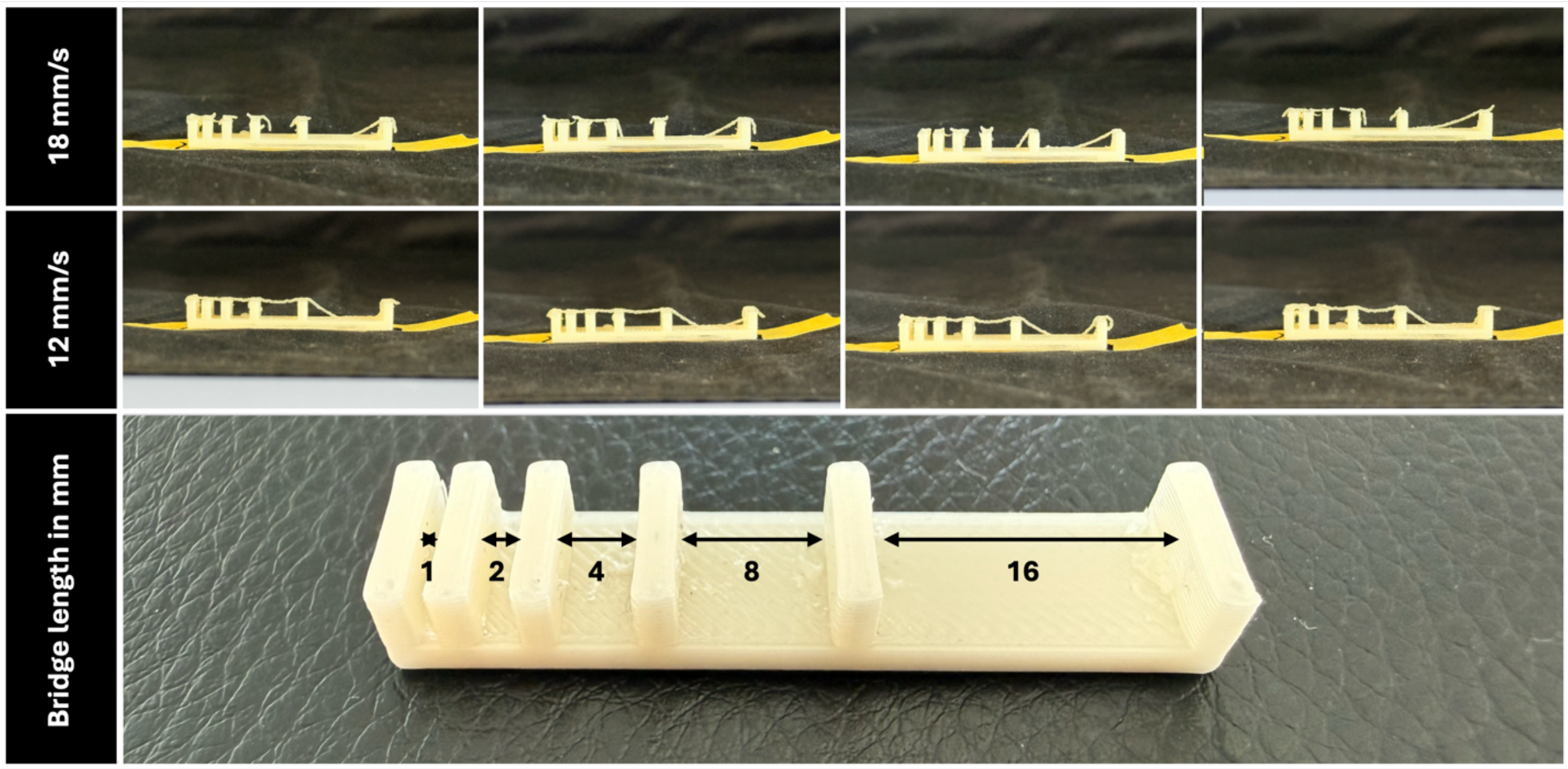


S3: Filament collapse test ground control for 120 kPa and speeds of 18 mm/s and 12 mm/s, with gaps of 1, 2, 4, 8 and 16 mm distance and n = 4 attempts per condition.
